# Who is a scientist? The relationship between counter-stereotypical beliefs about scientists and the STEM major intentions of Black and Latinx male and female students

**DOI:** 10.1186/s40594-021-00288-x

**Published:** 2021-04-07

**Authors:** Ursula Nguyen, Catherine Riegle-Crumb

**Affiliations:** Department of Curriculum and Instruction, STEM Education, The University of Texas, 1912 Speedway, Stop D500, Austin, Texas 78712, USA

**Keywords:** STEM counter-stereotypes, STEM beliefs, STEM majors, Gender, Race/ethnicity

## Abstract

**Background:**

Despite the diverse student population in the USA, the labor force in Science, Technology, Engineering, and Mathematics (STEM) does not reflect this reality. While restrictive messages about who belongs in STEM likely discourage students, particularly female and minoritized students, from entering these fields, extant research on this topic is typically focused on the negative impact of stereotypes regarding math ability, or the existence of stereotypes about the physical appearance of scientists. Instead, this study builds on the limited body of research that captures a more comprehensive picture of students’ views of scientists, including not only the type of work that they do but also the things that interest them. Specifically, utilizing a sample of approximately 1000 Black and Latinx adolescents, the study employs an intersectional lens to examine whether the prevalence of counter-stereotypical views of scientists, and the association such views have on subsequent intentions to pursue STEM college majors, varies among students from different gender and racial/ethnic groups (e.g., Black female students, Latinx male students).

**Results:**

While about half of Black and Latinx students reported holding counter-stereotypical beliefs about scientists, this is significantly more common among female students of color, and among Black female students in particular. Results from logistic regression models indicate that, net of control variables, holding counter-stereotypical beliefs about scientists predicts both young men’s and women’s intentions to major in computer science and engineering, but not intentions to major in either physical science or mathematics. Additionally, among Black and Latinx male students, counter-stereotypical perceptions of scientists are related to a higher likelihood of intending to major in biological sciences.

**Conclusions:**

The results support the use of an intersectional approach to consider how counter-stereotypical beliefs about scientists differ across gender and racial/ethnic groups. Importantly, the results also suggest that among Black and Latinx youth, for both female and male students, holding counter-stereotypical beliefs promotes intentions to enter particular STEM fields in which they are severely underrepresented. Implications of these findings and directions for future research, specifically focusing on minoritized students, which are often left out in this body of literature, are discussed.

## Introduction

Increasing the size of the workforce in Science, Technology, Engineering, and Mathematics (STEM) fields in the USA remains a pressing national priority.^1^ As such, researchers and policymakers continue to raise concerns about the need to attract and retain more students in STEM majors in college, particularly female and minoritized students given both historical and contemporary patterns of underrepresentation (National Science Board [NSB], National Science Foundation [NSF], 2019). Yet research indicates that many young people may be deterred from pursuing STEM fields due to prominent stereotypes regarding who best fits and belongs in such fields. Broadly speaking, this research generally falls into one of two related but distinct categories: studies that examine stereotypes about presumed gender or racial/ethnic differences in innate abilities in STEM fields (Beasley & Fischer, 2012; Shapiro & Williams, 2012), and studies that focus on stereotypical images or perceptions of scientists (Chambers, 1983; Cheryan et al., 2013). While research within the first category has been quite prolific in the past several decades (e.g., research on stereotype threat), research within the second category is less common.

Further, research on young people’s images or perceptions of scientists primarily focuses on issues of physical appearance (Chambers, 1983; Finson et al., 1995), and rarely examines whether and how individuals’ perceptions of scientists are linked to actual intentions to pursue STEM fields (Nassar-McMillan et al., 2011; Starr, 2018). Our study is purposively designed to address both limitations. Specifically, we utilize survey measures to capture a more comprehensive sense of students’ views of scientists, including not only the type of work that they do but also the types of things in which they are interested; we subsequently examine whether and how students’ views of scientists shape their intended pursuit of STEM fields in college. In doing so, we also deviate from the typical focus on how stereotypes deter or impede students’ STEM-related choices, and instead focus on the potential for counter-stereotypical perceptions of scientists (i.e., perceptions that scientists are multi-faceted individuals with a variety of interests and talents who do not work in isolation) to positively motivate students’ future plans. And given that educational and career plans begin to form well before college entry, with strong predictive power for actual choices as young adults, we focus on the perceptions of scientists held by adolescents as they transition into high school (Bandura et al., 2001; Eccles & Roeser, 2011; Morgan et al., 2013).

Finally, our study contributes new knowledge to the field through an explicit focus on Black and Latinx students. Despite the fact that the student population in US public schools is becoming increasingly more racially/ethnically diverse (de Brey et al., 2019), most of the research on STEM stereotypes focuses on predominantly White student populations. By contrast, we utilize longitudinal data collected from a sample of approximately 1000 Latinx and Black youth from a very large, urban public district located in the US Southwest to investigate the beliefs about scientists held by Black and Latinx students in middle school, and then examine whether and how these beliefs predict their college major intentions reported in high school. In doing so, our study is also informed by the insights of equity scholars who point out what can be lost when research assumes homogeneity or consistency within either gender or racial/ethnic groups (Collins, 1998; Ireland et al., 2018; Ong et al., 2011). Specifically, scholars applying an intersectional lens in STEM education research have articulated how race and gender are interlocking social systems that ultimately converge to create positions of power and privilege for White males, but also create distinct positions for marginalized groups at different points of intersection that need to be recognized; for example, the experiences and viewpoints of Black females should not necessarily be viewed as parallel to others who share their race or others who share their gender (Ireland et al., 2018; Ong et al., 2018; Rainey et al., 2018). Therefore, our study examines the perceptions of scientists held by different marginalized gender and racial/ethnic groups (e.g., Black female students, Latinx male students), and the impact such views may have on their subsequent intentions to pursue college majors.

Further, while the acronym of STEM has meaning and utility for many reasons, when asking students about their possible future paths of study, it is more informative to consider separate fields under that umbrella, particularly given women’s pronounced underrepresentation in some STEM fields (e.g., computer science), and not others (e.g., biological sciences). As such, we asked students to separately report the likelihood that they would major in each of the following: engineering, computer science, mathematics, physical sciences, and biological sciences. Our multivariate models also control for factors known to predict STEM intentions, including academic achievement and self-perceived science performance, in order to test the robustness of any observed association between perceptions of scientists and students’ future plans. Overall, our results draw attention to the STEM-related perceptions and expectations of youth who are too often marginalized in STEM fields and also relatively absent in research on STEM fields.

## Literature review

### Prior research on perceptions of scientists using the Draw-A-Scientist Test: insights and limitations

As mentioned above, research on young people’s images or perceptions of scientists predominantly focuses on physical appearances. Specifically, over the past several decades, the Draw-A-Scientist Test (DAST, developed by Chambers, 1983) as well as other modified versions (e.g., DAST-Checklist and Enhanced DAST) have been widely used as the primary instrument to examine students’ perceptions of scientists across many educational levels, including *elementary* (e.g. Chambers, 1983; Fung, 2002; Monhardt, 2003; Newton & Newton, 1998), *secondary* (e.g. Farland-Smith, 2009a; Hillman et al., 2014; Laubach et al., 2012), and *college or university* (Thomas et al., 2006). Generally, studies employing a version of the DAST instrument evaluate students’ drawings of scientists on the presence of stereotypical indicators. Initially, Chambers (1983) identified seven stereotypical indicators, including whether the scientist is wearing a lab coat, and whether the scientist has eyeglasses. Finson et al. (1995) created a checklist (DAST-C) to include additional indicators, including the gender (i.e., male), age, and race of the scientist, and whether they work outdoors. Overall, the body of research utilizing DAST illustrates students’ tendency to draw a stereotypical scientist that is White, male, wears eyeglasses and a lab coat, and works indoors or in a laboratory (Chambers, 1983; Christidou et al., 2016; Farland-Smith, 2009b; Finson et al., 1995; Fung, 2002; Hillman et al., 2014; Laubach et al., 2012; Monhardt, 2003; Newton & Newton, 1998; Shin et al., 2015; Thomas et al., 2006).

Regarding whether there are gender differences in who holds more or less stereotypical views of scientists, research using the DAST has found evidence that boys portrayed scientists with more stereotypical indicators in their drawings than girls (Christidou et al., 2016), and that girls tend to draw more female scientists than male students (Chambers, 1983; Christidou et al., 2016; Farland-Smith, 2009b; Thomas et al., 2006). Yet a recent meta-analysis found that over the last several decades, the percentage of students drawing female scientists has increased substantially across different age groups as well as genders (Miller et al., 2018). This offers promising evidence of a potential shift away from the prominence of explicit stereotypical depictions of scientists among contemporary generations of young people.

While certainly informative, the research literature using the DAST instrument nevertheless has many limitations. First, the DAST instrument has primarily been used descriptively, to provide an overview of how young people perceive scientists. As such, research has not considered whether stereotypical views of scientists as presumably captured by this instrument predict students’ science-related attitudes or behaviors. Thus, while research using the DAST is often motivated by the argument that narrow conceptualizations of scientists, particularly as White males, will deter the STEM interests and ambitions of young people, particularly those who are not White males, empirical studies do not actually test this supposition. Relatedly, the extant literature using the DAST is primarily limited to predominantly White samples, offering little insights into the views of minoritized youth (for notable exceptions see the following: Finson, 2003; Laubach et al., 2012; Monhardt, 2003).

Finally, while it does not escape us that the appeal of DAST lies in its feasibility to be readily employed as a tool to measure young children’s views of scientists, as it requires no writing, the findings may not fully capture the stereotypes students hold about scientists. Particularly, DAST may be capturing students’ awareness of stereotypical images of scientists’ physical appearance, as they see in the media (e.g., movies, cartoons, video games). That is, DAST and related instruments capture students’ broad perceptions of how scientists often *look*, and thus may be very applicable for capturing gender and even racial stereotypes, but otherwise rather limited.

### Broadening the measurement of perceptions of scientists: empirical evidence

There is a small body of extant research that moves beyond capturing perceptions of the physical appearance of scientists to instead provide a more comprehensive view of common conceptions of scientists by utilizing scales in survey research. For example, some research finds that students associate scientists with having high intellectual abilities, lacking interpersonal skills, working alone in laboratories, and possessing undesirable physical and personality traits (Beardslee & O’Dowd, 1961; Carli et al., 2016; Cheryan et al., 2013; Ehrlinger et al., 2018; Wyer et al., 2010). Correspondingly, some studies find that holding narrow stereotypical views of scientists is associated with lower levels of interest in pursuing STEM fields (Cheryan et al., 2013; Ehrlinger et al., 2018).

Yet, on the other hand, a handful of studies also provide evidence that many students have non-stereotypical views about scientists, and in turn, have favorable views about scientists and their scientific careers that could be considered counter-stereotypical in some ways (DeWitt et al., 2013; Fraser, 1978; Garriott et al., 2017; Nassar-McMillan et al., 2011; Schibeci, 1986; Smith & Erb, 1986; Wyer, 2003; Wyer et al., 2010). For example, in a study of about 1000 college students, Wyer et al. (2010) found that although students generally agreed that scientists are highly intelligent and work-oriented, they also agreed that scientists can have interpersonal competencies (e.g., cooperative, collaborative, family oriented, etc.). In a sample of over 9000 elementary students in England, DeWitt et al. (2013) observed that students overall hold positive views about scientists, including viewing their work as exciting and making a difference in the world. Moreover, while the undergraduate women in Starr’s study (2018) agreed with the genius stereotype scale (e.g., scientists are naturally very intelligent and obsessed with computers), they did not agree with the nerd stereotype scale (e.g., socially awkward, unattractive, introverted, etc.) about STEM workers.

Considering the aforementioned studies, this body of literature demonstrates that when using a more multidimensional scale that captures perceptions of scientists’ work activities, personal characteristics, and interests, there is evidence that recent cohorts of young people hold views of scientists that run counter to many prominent stereotypes. Further, some studies find that counter-stereotypical views are associated with an increased likelihood of reporting career or major selections in STEM fields (Cheryan et al., 2013; Erb & Smith, 1984; Nassar-McMillan et al., 2011). For example, Wyer (2003) observed that undergraduate students’ positive views of scientists were associated with multiple measures of STEM persistence (e.g., commitment to major, commitment to career, and advanced degree aspirations).

Additionally, among the few studies that consider potential differences in the perceptions of female students and male students (e.g., Nassar-McMillan et al., 2011; Schinske et al., 2015; Smith & Erb, 1986; Wyer, 2003), the evidence generally finds more gender similarities than differences. For instance, in a sample of undergraduate students, both men and women rated scientists similarly on agentic and communal traits (Carli et al., 2016). Similarly, Wyer (2003) observed no gender differences among undergraduate STEM students’ positive views of scientists and engineers. Yet as with DAST research reviewed above, we note that research utilizing scales to capture students’ perceptions of scientists in a more comprehensive way is nevertheless focused on predominantly White samples, not considering the beliefs of Black and Latinx students. This is a serious omission in the literature given that students of color comprise the majority (51%) of K-12 public school students (de Brey et al., 2019), and that focusing on White samples continues to privilege their viewpoints while silencing those of students from minoritized populations. Indeed, race and gender scholars highlight how research should not only include diverse samples but also attend to students’ intersectional identities, as the views and experiences of students from different gender and racial/ethnic groups are often unique or divergent from one another (Ong et al., 2018).

## Current study

Our study builds on the limited extant research that examines the perceptions that young people hold about scientists that go beyond ideas about physical appearance, to encompass the skills and interests that scientists have in addition to the work they perform. Specifically, informed by some promising findings that counter-stereotypical beliefs may be relatively common among contemporary cohorts, our study advances the literature by examining both the presence and potential future impact of holding strong counter-stereotypical beliefs.

The conceptual framework of our study is informed by social psychological theories which posit that when individuals are making decisions about the educational and occupational fields that they want to pursue, they consider the perceived fit between attributes of those in these fields and themselves (Diekman et al., 2017; Supeli & Creed, 2014). Specifically, as articulated by goal congruency theory, young peoples’ perceptions of the type of work and the characteristics of workers in different fields will shape their decisions; to the extent that those perceptions seem congruent or consistent with aspects of themselves, they will be more likely to want to enter a field (Diekman et al., 2010). As further articulated by Cheryan et al. (2015), young people’s choices to pursue STEM fields are shaped by perceptions of the culture of STEM fields, which includes perceptions of the common characteristics of the people in such fields, including their values and interests, and the type of work that they do. To the extent that young people hold stereotypical views that are very narrow and specific, this is likely to deter their intentions to enter such fields. Yet on the other hand, if students hold counter-stereotypical beliefs, such that they view scientists and their work as broader and more multidimensional, then they may be more likely to see themselves as belonging in such fields.

Thus, our study will specifically address the supposition that young people who hold counter-stereotypical views of scientists will be more likely to intend to pursue STEM fields than those who do not hold such views. In doing so, we further advance research on this topic through an explicit focus on Black and Latinx students, which represents a significant departure from the bulk of extant research that utilizes predominantly White samples. Additionally, we rely on intersectionality as a conceptual and methodological framework that calls attention to how both race and gender are interlocking social systems that create unique social positions and experiences for individuals within different race and gender groups (Collins, 1998; Ireland et al., 2018; Ong et al., 2011). And while this intersection of inequality undoubtedly preserves the power and privilege of White males, an intersectional lens can also highlight instances where one axis of stratification (i.e., gender or race) is more pronounced than the other. For example, while male students of color face many obstacles of discrimination and bias in STEM fields, they have similar rates of declaring STEM majors in college as White men (Garrison, 2013; Riegle-Crumb & King, 2010; Xie et al., 2015), which suggests that the social construction of STEM fields as masculine may work at least partly to advantage men of color in ways that simultaneously exclude women of color. Yet an intersectional lens acknowledges that a simple ‘additive’ approach to inequality (i.e., assuming that minoritized females always occupy the lowest social position as a consequence of their race and gender) risks missing important and distinct experiences of different groups. Indeed, prior research finds that compared to their White peers, Black female students are less likely to endorse traditional gendered stereotypes about male students’ presumably higher innate math ability, report comparatively higher levels of math self-efficacy, and more interest in pursuing STEM fields (Hanson, 2004; O’Brien et al., 2015). These studies offer empirical evidence of the insights regarding students’ STEM-related beliefs that can be gained when research moves beyond a focus on aggregate gender or racial differences. Therefore, we will examine whether both the prevalence and predictive power of counter-stereotypical beliefs vary between students belonging to different gender and racial/ethnic groups.

Additionally, our study is informed by developmental psychological theories which note that adolescence is a critical stage when young people are starting to decide who they want to become, and as such are developing their future career and postsecondary aspirations (Bandura et al., 2001; Eccles & Roeser, 2011). In particular, research on STEM trajectories has found that the future plans and expectations that form while students are in high school are highly predictive of the college major and occupations they actually enter later (Morgan et al., 2013). Yet research on students’ views of scientists often focuses on either young children at the elementary age (i.e., most of the DAST studies), or students already in college. While the former may be too early to validly assess college and career expectations, on the other hand, studies of college students’ stereotypical views about scientists may be capturing students’ beliefs too late (i.e., after they have already decided whether they can see themselves as scientists). Therefore, we measure students’ beliefs about scientists well before they actually enter college to better understand their potential impact on subsequent STEM-related choices.

Relatedly, in contrast to the cross-sectional design of most prior work on this topic, our study is longitudinal (as described in more detail below), following students across the transition from middle school to high school to examine how beliefs held during the former period predict the future expectations reported during the latter. In doing so, we recognize the importance of disaggregating the broad category of STEM to examine students’ expectations of majoring in different domains, including those that remain highly male-dominated (e.g., computer science), and those that are recently trending towards female-dominated at the undergraduate level (e.g., biological sciences). Finally, our analyses incorporate measures of students’ social and academic background, to ensure that the associations observed between beliefs about scientists and future intentions to pursue different STEM fields in college are robust to potential confounding variables.

Specifically, utilizing a large sample of students of color in one school district, we ask the following questions:

1. To what extent do Black and Latinx students hold counter-stereotypical beliefs about scientists, and does this vary by gender?
2. Does holding counter-stereotypical beliefs predict Black and Latinx students’ intentions to major in different STEM domains (e.g., biological sciences, computer science), and do patterns vary by gender?

## Methods

### Data and sample

The research team collected data from a very large urban school district (about 200,000 students total) as part of the Broadening Science in School Study (BSSS). The school district is located in the south-western United States in a city whose labor force includes industries in multiple STEM fields, including chemical and technological industries. The school district is mostly comprised of students of color, with approximately 70% Hispanic/Latinx students and approximately 25% Black students. Further, more than 75% of students qualify for free or reduced lunch, indicating economic disadvantage.

Over the course of several years, the BSSS research team collected cross-sectional survey data from several cohorts of middle-school students, which included items related to attitudes about science, as well as administrative data, including students’ test scores, and transcripts (Blanchard Kyte & Riegle-Crumb, 2017; Riegle-Crumb & Morton, 2017). In this study, we utilize longitudinal data from a sub-sample of the larger project, a cohort of 8th grade students in the district who completed a survey in the 2012–2013 academic year, and who were followed into high school and later completed a follow-up survey (*n* = 1108 students from 216 eighth-grade science classrooms across 30 middle schools). Students’ race/ethnicity and gender were provided by the district via administrative files and utilized to determine the gender and racial/ethnic composition of the sample as follows: 469 Latinx female students, 107 Black female students, 434 Latinx male students, and 98 Black male students. Consistent with district enrollment, our sample is predominantly Latinx.

### Dependent variables

The dependent variables are constructed from items in the high school survey that asked students about their likelihood of majoring in each of five separate STEM fields: biological sciences, physical sciences, mathematics, computer science/technology, and engineering. Thus, we have five different outcomes to capture students’ STEM intentions—one for each field. The original response categories ranged from 1 (not at all likely) to 5 (very likely), with 3 corresponding to a neutral response. Exploratory analyses revealed that responses were not normally distributed, and instead were significantly and highly positively skewed, as most students reported a low likelihood of majoring in these STEM fields. Therefore, we dichotomized the dependent variables, such that 1 represents that student responded that they were likely (score of 4 or 5) to major in a particular STEM field, and 0 represents that the student responded they were not likely (score of 3 or below) to major in that particular field.^2^

While initial descriptive analyses in Table 1 reveal that only 15% of adolescent students reported they would likely major in biological sciences and physical sciences, about twice as many said they would likely major in mathematics (30%), computer sciences (27%), and engineering (34%). Moreover, students’ intentions are related across some STEM fields. In particular, students’ intentions to major in biological sciences are strongly correlated to their intentions to major in physical sciences (*r* = 0.75, *p* < 0.001), and likewise, students’ intentions to major in computer sciences are also strongly related to their engineering major intentions (*r* = 0.59, *p* < 0.001).

Further, consistent with national data on gender differences in bachelor’s degree attainment (NSB, NSF, 2019), we also observe distinct gender differences in certain STEM fields, such that a significantly lower proportion of girls reported that they expected to major in computer science (20.5%, *χ*^2^ = 27.73, *df* = 1, *p* < 0.001), and engineering (19.1%, *χ*^2^ = 124.29, *df* = 1, *p* < 0.001), compared to their male counterparts, (34.6% and 50.9%, respectively). These gender disparities were comparable for both Latinx and Black students.

### Independent variables

#### Counter-stereotypical beliefs about scientists

The key independent variable for our study is a scale that captures students’ counter-stereotypical beliefs about scientists. To measure adolescent students’ perceptions of scientists beyond physical traits, the research team adapted five items from previous related studies that surveyed elementary students (DeWitt et al., 2011) and college students (Wyer et al., 2010). Two items ask about the type of work that scientists do: “Scientists usually work alone in labs” and “Scientists can work in teams or groups”; while three items capture views about scientists’ interests and personal characteristics: “Most scientists are geeks or nerds,” “People that are good at science cannot be good at other things, like sports or art,” and “Scientists are curious and creative people.” Each of these items had response categories ranging from 1 (Strongly Disagree) to 4 (Strongly Agree); items with a negative valence (e.g., *Most scientists are geeks or nerds*) were reverse-coded so that for all items, a higher score represented a more positive or counter-stereotypical view. Using the Skewness-Kurtosis test for normality (via Stata statistical software), we determined that the distribution of all five items significantly deviated from the normal distribution regarding both values of skewness and kurtosis. Specifically, consistent with some prior research discussed above (Garriott et al., 2017; Nassar-McMillan et al., 2011), the items were all negatively skewed, such that overall students held somewhat positive views about scientists. Therefore, we dichotomized each item, such that 1 represented strongly agreeing with a counter-stereotypical statement about scientists (score of 4 or strongly agree) and 0 represented a score of 3 or below.^3^

To determine the factor structure underlying the five items related to counter-stereotypical beliefs about scientists and establish its validity, we randomly divided our sample into two separate and equal-sized groups to conduct exploratory and confirmatory factor analyses (DeCoster, 1998; Fabrigar et al., 1999). The exploratory factor analysis (EFA) using a principal factors extraction method was conducted with the first sub-set of data, representing half of our dataset (*N* = 554). This produced a one-factor solution, as indicated by the inspection of the scree plot test and eigenvalues (Costello & Osborne, 2005), that explained about 41% of the variance. The Kaiser-Meyer-Olkin measure of 0.72 indicated an acceptable sampling adequacy (Kaiser, 1974). Additionally, all items loaded onto the single factor, with factor loadings ranging from 0.58 to 0.71, and communalities ranging from 0.33 to 0.51. These moderate to strong factor loadings and our conceptualization of students’ counter-stereotypical beliefs about scientists suggest that all five items be retained (Costello & Osborne, 2005). Subsequently, a confirmatory factor analysis (CFA) was conducted to validate the structure of the factor with the second half of our sample (*N* = 554). The fit statistics indicated an adequate model fit, including a Root Mean Square Error of Approximation (RMSE A) of 0.08 (where values less than or equal to 0.08 indicate an acceptable fit, and 90% CI is [0.05,0.11]), a comparative fit index (CFI) of 0.94 (where values greater than 0.90 indicate an acceptable model fit) and a Standardized Root Mean Squared Residual (SRMR) of 0.04 (where values less than or equal to 0.08 indicate a good fit) (Hu & Bentler, 1999; Kline, 2015). While the chi-squared statistic for the CFA model was significant (*χ*^2^ = 21.67, *df* = 5, *p* < 0.001), this statistic is known to be sensitive to large sample sizes such as ours (Hair et al., 2010). The standardized factor loadings ranged from 0.41 to 0.54, with a Cronbach’s alpha of 0.60, which is somewhat low but still within the acceptable range (Bagozzi & Yi, 1988; Hair et al., 2010).

Finally, to create the scale used in the analyses presented here, students’ responses across all items were averaged, so that higher scores on the scale capture holding more counter-stereotypic beliefs about scientists, while lower scores represent the opposite. Put simply, our scale is a measure of strong counter-stereotypical beliefs about scientists, as it captures beliefs that are not simply neutral opinions of scientists, but rather views of scientists that are the anti-thesis of traditionally narrow and restrictive stereotypical images (e.g., scientists are geeks or nerds, and scientists work alone in a lab). We report gender and racial/ethnic differences in counter-stereotypical beliefs in the results section.

#### Additional individual (student-level) variables

While national trends show that students of color remain disproportionately represented among STEM college major entrants (NSB, NSF, 2019), we distinguish between those students who are Latinx (or Hispanic) and those who are Black. As noted earlier, this sociodemographic variable along with students’ gender was provided by the district through administrative files. However, we include them in our models as we do not try to assume students of color to be a homogenous group, and therefore allow for potentially differing effects to be observed.

We also include variables to control on student characteristics that prior research has shown may be related to decisions to pursue STEM majors, including social class and self-perceptions of performance (Chen, 2009; Engberg & Wolniak, 2013; Maple & Stage, 1991; Wang, 2013). As a proxy for social class, we include a measure of mother’s highest educational level (Ridolfo & Maitland, 2011); this was created from a student-reported survey item and dichotomized so that 1 indicates that the mother completed a Bachelor’s degree or higher and 0 indicates that the mother did not complete a Bachelor’s degree. We capture whether students have a high self-perception of science performance via a survey item asking students’ level of agreement with the statement that they “usually do well in science”; original response categories ranged from 1 (Strongly Disagree) to 4 (Strongly Agree). To address its significant skewness (value of − 0.66, with kurtosis = 3.19), this variable was dichotomized so that 1 represents strong agreement and 0 reflects the reverse.

Additionally, as previous studies have found that achievement is significantly related to students’ interest in STEM majors (Lichtenberger & George-Jackson, 2013; Wang, 2013), we include students’ scores on a standardized math test administered by the district as a control. This is a continuous variable that originally ranged from 0 to 52 but was standardized and ranges from − 1.59 to 2.02. We note that we have information on mathematics (but not science) achievement, as mathematics tests are administered annually in the district as part of school accountability. However, prior research has found a strong correlation between mathematics and science achievement (Else-Quest et al., 2013; Maerten-Rivera et al., 2010; Wang, 2005), and math achievement has also been found to positively predict STEM outcomes such as choice of major (Wang, 2013).

Table 1 shows the means (or percentages) and standard deviations for each of the predictor variables for the entire sample as well as for each gender. Table 4 in the Appendix shows the correlations between predictor variables in our models. Additionally, we checked for multicollinearity by examining variance inflation factor (VIF) and tolerance values. VIF values ranged from 1.00 to 1.11, all well below 10, while tolerance values ranged from 0.90 to 1.00 (and all above 0.10), indicating that multicollinearity is very low and not an issue at hand.

## Results

### Analytic approach

In this section, we first present descriptive results, that is, means on the scale measuring students’ counter-stereotypical beliefs about scientists. Specifically, to address the first research question, we examine whether there are mean gender differences, and further, whether there are racial/ethnic differences within gender groups.

Subsequently, to examine whether students’ beliefs about scientists predict intentions to major in STEM fields, we utilize single-level binary logistic regression models given that our five dependent measures, intentions to major in each of five different STEM fields, are dichotomous variables. Although our data is clustered, analyses of variance across levels for our models revealed very little to no variation at levels 2 (classroom) and 3 (school). Specifically, the intraclass correlation values at level 2 ranged from 0 to 0.101, and values at level 3 ranged from 0 to 0.032, thus indicating the use of single-level logistic regression models as more appropriate (Bryk & Raudenbush, 1992).^4^

We conduct separate analyses by gender to more easily observe whether the patterns observed are similar or different for adolescent girls of color and for boys of color. We note that our intent is not to deliberately search for a more parsimonious model, but rather remain conservative in how we present the findings surrounding our key independent variable of our study. Specifically, the model-building approach we employ is driven primarily by our understanding of empirically based research on student sociodemographic and academic factors related to students’ intentions to major in STEM fields (Lichtenberger & George-Jackson, 2013; Maple & Stage, 1991; Wang, 2013). As such, we perform stepwise models where the first (baseline) model includes only the focal independent measure of students’ beliefs about scientists, and then add all control variables in the second model to test the robustness of the association. Finally, within a sample comprised of students of color, we do not assume that beliefs about scientists necessarily predict future intentions in the same way for Latinx and Black youth; as such, in model 3, we add interactions between students’ race/ethnicity and their beliefs. We report logistic regression coefficients from models in the tables but translate key results into odds ratios (by exponentiating the coefficients) for ease of interpretation.

Along with the results of the logistic regression models, Tables 2 and 3 also present model fit statistics. Specifically, the Hosmer-Lemeshow goodness-of-fit statistic was used to assess the overall fit of individual models, and the results indicate an overall good model fit for each of the logistic regression models (Archer & Lemeshow, 2006; Long & Freese, 2006). We also include Akaike information criterion (AIC), Bayesian information criterion (BIC), and likelihood ratio (LR) test statistics for comparison between models.

### Counter-stereotypical beliefs about scientists among students of color

**Fig. 1:**
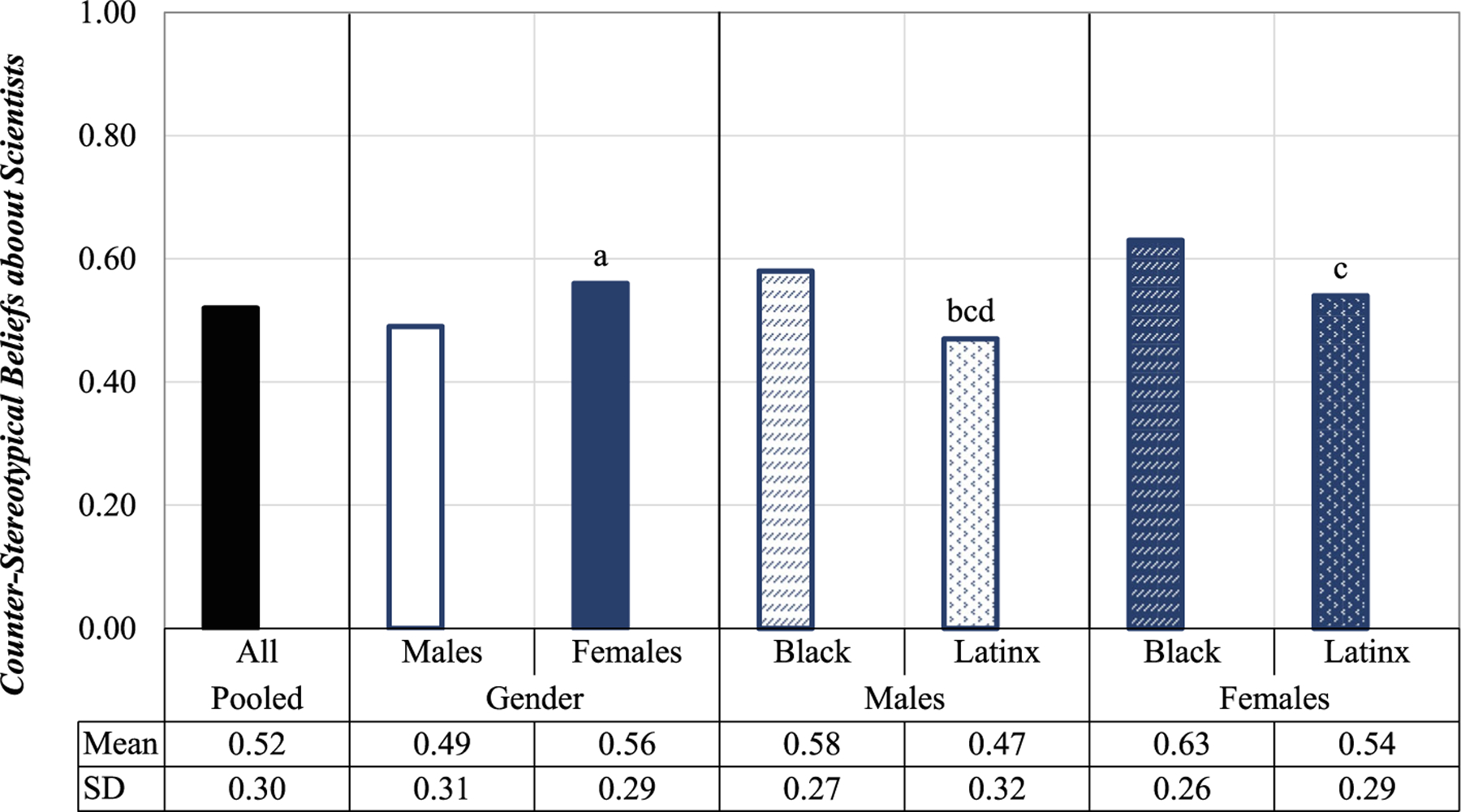
Counter-stereotypical beliefs about scientists. A higher value on the scale indicates that students report more counter-stereotypical views about scientists. The “a” indicates that the mean for females is statistically significantly different from that of males (*p* < 0.001). Similarly, “b” indicates that the mean for Latinx males is significantly different from that of Black males (*p* < 0.01), “c” indicates that means for Latinx students (of both genders) are significantly different from that of Black females (*p* < 0.001), and “d” indicates that mean of Latinx males is significantly different from that of Latinx females (*p* < 0.001)

In Fig. 1, we present the means and standard deviations of the scale measuring students’ counter-stereotypical beliefs about scientists separately by gender. Additionally, we also provide separate means and standard deviations for each racial/ethnic group within each gender. Keeping in mind that the scale ranges from 0 (low) to 1 (high), as shown in the *y*-axis, the results indicate that adolescent girls of color in our sample hold more counter-stereotypical beliefs about scientists (mean = 0.56) than boys of color (mean = 0.49). We note that this gender difference (*t* = 3.88, *p* < 0.001) is rather small, at approximately a quarter of a standard deviation. Additionally, Latinx adolescent males held lower counter-stereotypic views of scientists (mean = 0.47) than all other groups of students (compared to Black males, *t* = 3.40, *p* < 0.01; compared to Black females, *t* = 5.21, *p* < 0.001; and compared to Latinx females, *t* = 3.56, *p* < 0.001). Black girls (mean = 0.63) held more counter-stereotypical beliefs than Latinx youth of either gender (compared to Latinx males, *t* = 5.21, *p* < 0.001; and compared to Latinx females, *t* = 3.04, *p* < 0.01).^5^

### Female students’ intentions to major in STEM fields

Table 2 shows the results of logistic regression models predicting adolescent girls’ intentions to major in the separate STEM fields. Beginning with models for the biological sciences, in the baseline model, holding more counter-stereotypical beliefs is significantly associated with a higher likelihood of intending to pursue a major in this domain. Specifically, increasing from 0 to 1 on the scale is associated with an increase in the odds of majoring in biological sciences by a factor of approximately 2.38. Yet, this association is no longer statistically significant with the addition of control variables in model 2 (and adding these variables improves model fit). In model 3, we add an interaction between beliefs about scientists and students’ race/ethnicity; the coefficient is not statistically significant (and does not improve model fit).

Continuing on with models predicting girls’ intention to major in physical sciences, the results reveal that there is not a statistically significant association between beliefs about scientists and the outcome (see models 4 and 5); nor is there a significant interaction between race/ethnicity and beliefs (model 6). We see a similar pattern of null results for models predicting girls’ intentions of majoring in mathematics (see models 7, 8, and 9). In both sets of models, adding the control variables improves model fit, while adding the interaction terms does not.

The last two sets of models show the relationship between counter-stereotypical beliefs about scientists and girls’ intentions to major in computer science and engineering, respectively. The results reveal a positive and statistically significant relationship for both STEM domains. Specifically, at baseline, increasing from 0 to 1 on the scale is associated with an increase in the odds of intending to major in computer science (model 10) by a factor of 2.39, and an increase by a factor of 3.74 in the odds of intending to major in engineering (model 13). Further, the inclusion of student-level control variables in the full model does not improve model fit and leaves the association relatively unchanged, such that among girls of color, the odds of intending to major in computer science (model 11) and engineering (model 14) increases by a factor of 2.59 and 3.49, respectively, with an increase from 0 to 1 on the scale measuring counter-stereotypical views of scientists. The interaction terms between beliefs and race/ethnicity are not significant for either domain (see models 12 and 15), indicating that these relationships are statistically comparable for Black and Latinx girls.

Regarding other variables in the models for each outcome, we note that for three STEM outcomes (i.e., biological sciences, physical sciences, and mathematics), girls’ self-perceived science performance is a strong and significant predictor of their intentions to major in these respective fields. Specifically, girls who have high self-perceptions of their science performance are about 1.80 times more likely to intend to major in biological sciences, physical sciences, and mathematics than those who had a lower self-perception of science performance. Yet the same is not true for their intentions to major in computer science and engineering, where null results are observed for high self-perceptions of science performance. Additionally, no other control variable is related to girls’ intentions to major in any of these STEM fields.

### Male students’ intentions to major in STEM fields

Next, we turn to Table 3, which shows the results for logistic regression models predicting adolescent boys’ intentions to major in each of five different STEM fields. Among our sample of adolescent boys of color, in the baseline model (model 1), intentions to major in the biological sciences were significantly and positively related to holding counter-stereotypical beliefs about scientists. This relationship remained robust, although somewhat weaker, with the addition of all control variables, which do not improve model fit (model 2); specifically, an increase from 0 to 1 on the scale is associated with an increase by a factor of 2.59 in the odds of intending to major in the biological sciences. The interaction between beliefs and boys’ race/ethnicity was not significant and does not improve model fit (model 3). That is, this relationship between boys’ intention to major in biological sciences and their counter-stereotypical beliefs about scientists is similar between Black and Latinx boys.

Moving to the next set of models, while holding more counter-stereotypical beliefs about scientists is associated with a significantly higher likelihood of intending to major in the physical sciences at baseline (model 4), this association is not robust to the inclusion of control variables (which improve model fit as seen in model 5), nor is there evidence of a significant interaction with race/ethnicity (model 6). Moreover, similar to the results for the models pertaining to girls’ intentions to major in mathematics discussed above, we also observe no significant relationship between boys’ intention to major in mathematics and their counter-stereotypical perceptions about scientists (models 7 and 8); nor is there a significant interaction by race/ethnicity (model 9).

Continuing on to results of analyses predicting boys’ intentions to major in computer science, counter-stereotypical beliefs about scientists are associated with their intentions to major in this STEM domain at the baseline (model 10); this association is significant with the inclusion of all control variables, which do not increase model fit (model 11). While at baseline, the odds of boys intending to major in computer science increases by a factor of 1.77 with an increase from 0 to 1 on the scale, at the full model (model 11), the odds of intending to major in computer science increases by a factor of 1.98.

A similar trend is observed from results predicting boys’ intention to major in engineering, such that at the baseline, increasing from 0 to 1 on the scale measuring counter-stereotypical beliefs is related to an increase in the odds of intending to pursue this particular major by a factor of 2.08 (model 13). Further, once all individual control variables are taken into account, an increase from 0 to 1 on the scale results in an increase by a factor of 2.29 in the odds of boys’ intentions to major in engineering (model 14). Again, similar to the results for girls, we observe no racial/ethnic interactions between boys’ counter-stereotypical views about scientists and their intentions to pursue computer science (model 12) and engineering majors (model 15).

Aside from our key independent measure of interest, only high self-perceived science performance is significantly associated with any of the outcomes. Yet even then, self-perceived science performance is only significant in models predicting boys’ intentions to major in physical sciences (where its inclusion improves model fit), such that those with a particularly high self-perception of science performance are 2.49 times more likely to intend to pursue a major in the physical sciences than their counterparts with lower self-perceived science performance. Taken together, these results reveal that similar to the results for the analyses for girls’ STEM major intentions, individual background characteristics such as prior math achievement and mother’s educational level are not significantly associated with boys’ intentions to major in STEM fields. On the other hand, students’ counter-stereotypical beliefs about scientists remained a robust and significant predictor of male students’ likelihood of intending to major in biological sciences, computer science, and engineering net of these variables.

## Discussion

Informed by research which finds that adolescence is a critical time for the crystallization of career aspirations (Bandura et al., 2001; Eccles & Roeser, 2011; Morgan et al., 2013), this study sought to investigate the potential impact of holding counter-stereotypical beliefs about scientists on students’ intentions to major in STEM fields. Moreover, while the views of students from nondominant communities are often missing from or treated as incidental to research on this broader topic, we focused explicitly on the views of Black and Latinx students, and considered whether and how their views subsequently predicted their intentions to major in five different STEM domains. Further, we considered whether patterns were similar or dissimilar by gender, motivated by the recognition of the interlocking connections between systems of race and gender inequality, and therefore the need to attend to students’ unique identities as a consequence of this intersection. In doing so, we make new contributions to this field of research, which still too rarely acknowledges the continued power and privilege of White males in STEM fields.

Our analyses examining Black and Latinx adolescents’ perceptions reveal that about half of the sample reported beliefs that could be considered counter-stereotypical. Although empirical research using diverse secondary student samples is sparse, our results are comparable with studies that have found that adolescents often have positive views about scientists and their work (Fraser, 1978; Garriott et al., 2017; Smith & Erb, 1986). Thus, despite the prevalence of negative, narrow, and distorted images of scientists often found in the media (e.g., shows such as *Big Bang Theory*), and related concerns about their potential influence on students’ perceptions of scientists (Song & Kim, 1999; Steinke et al., 2007), the present study demonstrates that contemporary youth, such as the Black and Latinx students that comprise our sample, hold perceptions of scientists that lean away from these traditional stereotypes.

Yet the data also suggest some notable differences along the lines of both gender and race/ethnicity. Specifically, girls of color tend to hold more counter-stereotypical beliefs about scientists than their male counterparts. This is consistent with the work of Schibeci (1986) and Christidou et al. (2016), which found that girls held more favorable views about scientists (or less stereotypical views about scientists) than boys. Further, in our sample, Black girls endorsed more counter-stereotypical views about scientists than Latinx youth of either gender. These results point to the importance of considering the heterogeneity of beliefs students possess about scientists, especially as the student population in public schools becomes more diverse (de Brey et al., 2019). Simply put, this study provides evidence of the nuanced ways in which counter-stereotypical perceptions of scientists appear at the intersection of students’ gender and race/ethnicity.

Moreover, the patterns revealed in our data may be indicative that positive role models in science, and STEM more broadly, have captured the attention of Black and Latinx students, particularly Black girls, such that they believe that scientists are multi-dimensional individuals, who can be smart and creative people with many diverse interests, including a curiosity about the world around them. While most of the research focusing on gender and racial stereotypes in STEM fields tends to focus on stereotypes about the presumed differences in skills and abilities between groups (which remains an important topic of study), we suggest that future research is also needed to understand how adolescents’ views of scientists are shaped, including investigating the kinds or sources of information that may be most salient in contributing to the somewhat broader views observed among girls of color, and Black girls in particular, in our sample. For example, we are beginning to see positive movements in social media, such as the hashtags #ThisIsWhatAScientistLooksLike and #ILookLikeAnEngineer, which demystify the everyday jobs STEM workers do, and may help adolescents form counter-stereotypical images of scientists.

Turning our attention to how these views shape later intentions to major in specific STEM fields, our results from the multivariate models indicate that counter-stereotypical beliefs about scientists do in fact matter for Black and Latinx students’ intentions to major in particular fields of STEM. Although consistent with prior evidence of the relationship between students’ views of scientists and their STEM-related aspirations (Cheryan et al., 2013; Starr, 2018; Wyer, 2003), our study stands out by paying particular attention to adolescents (rather than college students) who are Black and Latinx students (rather than predominantly White). Further, our results found overall similarities between the patterns observed for adolescent girls and boys. Specifically, we did not find robust evidence that counter-stereotypical views predicted intentions to major in either the physical sciences or mathematics for students of either gender. Perhaps the general parallel results observed for models predicting intentions to major in mathematics and physical science are a reflection of the strong overlap in content between these fields as well as students’ perceptions of the similarities between these STEM fields. Yet for both genders, those who endorsed counter-stereotypical views of scientists were more likely to intend to major in computer science as well as engineering in college; these results were robust to the inclusion of individual-level control variables.

Stepping back, it is interesting that holding broader and more positive views of scientists is important for predicting both boys’ and girls’ future intentions to pursue the fields of computer science and engineering. For girls in our sample, holding more counter-stereotypical beliefs is a positive predictor of intending to enter these two extremely male-dominated fields, perhaps because they work to outweigh concerns about gender-related norms and expectations. Yet our results also reveal that such beliefs may embolden the intentions to pursue these fields among boys in our sample (who on average report much higher expectations of majoring in these two fields than their female peers of color). As such, while these fields are normatively and stereotypically masculine, it is possible that relinquishing a narrow and restrictive view of scientists also allows adolescent boys of color to see themselves in fields that are currently predominantly White.

We did find some limited evidence suggesting differences in patterns by gender for models predicting intentions to major in the biological sciences, such that for male students, the effect of holding counter-stereotypical beliefs on intentions to major in this field was significant and remained robust even with the inclusion of control variables. Given that the biological sciences are approximately 60% female at the baccalaureate level, and this female advantage in biological science is found across all racial/ethnic groups (Cheryan et al., 2017; NSB, NSF, 2019), perhaps viewing scientists as more multi-dimensional and well-rounded people gives young men a boost to enter a field that is increasingly non-normative for their gender. Put differently, Black and Latinx boys are more likely to intend to major in biological sciences, a field where they are underrepresented relative to their female counterparts, when they endorse strong counter-stereotypical beliefs about scientists.

Finally, within our sample of students whose racial/ethnic backgrounds are traditionally underrepresented in STEM fields, our analyses did not identify any interactions between race/ethnicity and counter-stereotypical views on students’ intention to major in the different fields of STEM. This was true for both adolescent males and females of color. In other words, although Latinx and Black youth differed in their endorsement of counter-stereotypes about scientists, this difference in beliefs did not have an impact on their intentions to major in STEM fields. Given that both racial/ethnic groups are severely underrepresented across degrees in all STEM fields, it is encouraging that counter-stereotypical beliefs appear to similarly boost their expectations to enter some STEM fields.

### Limitations

Despite the new insights our findings contribute to this research area, we also note several limitations of our study. First, unpacking how and why counter-stereotypical beliefs about scientists play a significant role in predicting intentions to major in computer science and engineering is beyond the scope of this study. Second, while the alpha for our scale measuring counter-stereotypical beliefs is within the acceptable threshold (Bagozzi & Yi, 1988; Hair et al., 2010), nevertheless reliability should be interpreted with caution. Future research could build on our study and assess the internal structure and reliability of the counter-stereotypical beliefs scale when studying a different population of students. Additionally, future research, including qualitative studies, should ascertain how these beliefs are formed and how both female and male youth of color rely on these beliefs as they pursue STEM pathways.

Further, although STEM major intentions formed during high school have been shown to be highly predictive of subsequent choices (Morgan et al., 2013), nevertheless our study is not able to ascertain whether the students in our sample do actually translate their expectations into reality. Additionally, while we contribute to the need for more research on minoritized youth (i.e., Black and Latinx), at the same time, we cannot comment on the perceptions of scientists and related implications for youth from other racial/ethnic backgrounds. Moreover, we recognize that there are limitations in using categorical assessments of race and gender, as we have in the present study, rather than using measures of racial and gender identities or salience, which were not included in the student survey. Future research studies should strive to use measures that more authentically capture how students view their own identities.

Our study is also limited to one, albeit very large, public school district, and as such, may not have broad generalizability across other districts and states. Additionally, this school district is located in a city whose labor force includes corporations in various STEM industries, and thus it is possible that students there may be exposed to more broad social messaging about STEM and know more people that are STEM workers compared to other districts. Therefore, future research that includes students from many diverse districts and cities across the country could shed light on how prevalent counter-stereotypical views about scientists are among different contexts.

## Conclusion

Our study provides new insights into the perceptions and views about scientists held by a sample of Black and Latinx students, particularly, at the critical age of adolescence. While prior work provides well-documented evidence of students’ stereotypical images of scientists in the form of drawings of scientists, we note here the importance of attending to students’ notions of scientists that go beyond their physical appearance. That is, our study utilizes survey measures capturing students’ counter-stereotypic views about scientists, including their interests and work. Our findings suggest that Black and Latinx students do hold positive (i.e., counter-stereotypical) beliefs about scientists. Additionally, our results also point to the value of applying an intersectional lens when examining such beliefs to uncover the nuanced and complex ways in which youth of color endorse these beliefs. Namely, Black girls reported more strongly counter-stereotypical beliefs than their Latinx peers of both genders.

Delving into the relationship between these beliefs and future intentions to major in STEM fields, we observe a positive association between counter-stereotypical views of scientists and students’ intentions to major in particular fields of STEM, including computer science and engineering. We note that the strength of our longitudinal data lies in the ability to analyze high school students’ reported intentions to major in various STEM fields in relation to their counter-stereotypical beliefs held as middle school students, net of other factors. For both boys *and* girls of color, we observe the salience of such counter-stereotypical perceptions of scientists in their intentions to major in computer science and engineering.

### Implications

Our findings speak to the work that needs to occur in STEM classrooms, as well as changes in education policy. For the former, creating positive views of scientists in the minds of young people likely requires that they be able to see real scientists and engineers and also engage in work that is authentic to what they do. Interestingly, our results found that holding counter-stereotypical views about scientists predicted students’ intentions to major in computer science and engineering, and not either mathematics and physical sciences. This may be due to the prevalence of real-world applications of the work that computer science and engineers do. For instance, students have access to technology (e.g., laptops, cellphones, etc.) created by these workers and presumably use these technologies, such as apps and social media, in their everyday life. Perhaps making direct connections to the work that mathematicians and physical scientists do and the impact of their work on their world may provide students alternative images of these types of STEM workers.

Importantly, we also recognize that having positive images and beliefs about scientists is not at all sufficient to promote racial/ethnic and gender equality in representation in STEM fields. Rather, we recognize the power of structural inequality, such as systemic racism in the form of educational inequality and opportunities to pursue STEM, and the sexism and bias that creates chilly climates for women pursuing STEM (Cheryan et al., 2017; Morris & Daniel, 2008; Xie et al., 2015). Although our results suggest the relevance of counter-stereotypical perceptions at the individual level, we do not imply that students should become resilient to these forms of discriminations, nor should the work be left up to them to navigate these systems while challenging negative stereotypes about scientists. On the contrary, we believe that educational policies should acknowledge and deter inequality in K-12 education, beginning with ensuring all students participate in engaging and powerful mathematics and science instruction, so that these experiences can shape the beliefs students hold about scientists. Transforming educational experiences in mathematics and science for Black and Latinx students of all genders includes providing more resources to their schools and access to high-quality teachers and advanced STEM/mathematics course-taking. By actively working against educational inequality, these policies, in turn, can be consequential in changing students’ views of who scientists are and the work that they do. That is, policies that reduce educational inequality endured by students from nondominant communities, particularly young men and women of color, can also bring them closer to the line of work that scientists and engineers partake in, which can shape their beliefs about scientists and engineers, and, in turn, impact their future intentions to pursue STEM careers.

## Figures and Tables

**Table 1:** Descriptive statistics: dependent and control variables

	Pooled		Gender			
	*Mean (proportions)*	*SD*	Males		Females	
			*M*	*SD*	*M*	*SD*
**Dependent variables**						
Intentions to major in Biological Sciences	0.15		0.14		0.16	
Intentions to major in Physical Sciences	0.16		0.16		0.16	
Intentions to major in Mathematics	0.30		0.32		0.28	
Intentions to major in Computer Science	0.27		0.35		0.20	
Intentions to major in Engineering	0.34		0.51		0.19	
**Control variables**						
Race/ethnicity						
Black	0.19		0.18		0.19	
Latinx	0.82		0.82		0.81	
Mother’s highest levelof education						
Less than a Bachelor’s degree	0.84		0.81		0.86	
Bachelor’s degree or higher	0.16		0.19		0.14	
Standardized 8th grade math test score	− 4.49E−09	1.00	3.30E−02	0.99	− 3.04E−02	1.01
8th grade high self-perception of science performance	0.31		0.35		0.27	
*N*	1108		532		576	

**Table 2:** Results of logistic regression models predicting female students’ intentions to major in STEM Fields

Variables	Biological sciences			Physical sciences			Math			Computer Science			Engineering		
	Model 1	Model 2	Model 3	Model 4	Model 5	Model 6	Model 7	Model 8	Model 9	Model 10	Model 11	Model 12	Model 13	Model 14	Model 15
	Baseline	Full	Full + interaction	Baseline	Full	Full + interaction	Baseline	Full	Full + interaction	Baseline	Full	Full + interaction	Baseline	Full	Full + interaction
**Counter-Stereotypical Beliefs about Scientists**	0.869* (0.409)	0.584 (0.424)	− 0.493 (0.911)	0.498 (0.408)	0.206 (0.428)	0.389 (1.042)	0.516 (0.330)	0.419 (0.343)	− 0.303 (0.865)	0.871* (0.373)	0.953* (0.385)	0.659 (0.950)	1.318*** (0.392)	1.249** (0.404)	1.610 (1.084)
**Interaction**															
Beliefs × Latinx (ref = beliefs × Black)			1.346 (1.018)			− 0.218 (1.130)			0.844 (0.933)			0.347 (1.028)			− 0.418 (1.159)
**Controls**															
Latinx (ref = Black)		− 0.134 (0.290)	− 0.986 (0.692)		0.028 (0.306)	0.168 (0.792)		0.159 (0.256)	− 0.367 (0.627)		0.045 (0.278)	− 0.177 (0.712)		0.276 (0.295)	0.560 (0.848)
Mother’s highest level of education		0.285 (0.314)	0.301 (0.313)		− 0.141 (0.346)	− 0.144 (0.347)		− 0.456 (0.299)	− 0.444 (0.299)		0.071 (0.305)	0.079 (0.305)		0.023 (0.313)	0.013 (0.315)
Standardized 8th grade math test score		0.101 (0.113)	0.102 (0.113)		0.011 (0.113)	0.010 (0.113)		0.044 (0.093)	0.044 (0.094)		− 0.070 (0.103)	− 0.071 (0.103)		− 0.057 (0.104)	− 0.057 (0.104)
8th grade high self-perception of science performance		0.654** (0.245)	0.668** (0.245)		0.883*** (0.247)	0.881*** (0.247)		0.587** (0.210)	0.597** (0.210)		− 0.285 (0.247)	− 0.281 (0.247)		0.326 (0.236)	0.323 (0.237)
Constant	− 2.150*** (0.271)	− 2.134*** (0.387)	− 1.447* (0.623)	− 1.970*** (0.264)	− 2.098*** (0.396)	− 2.217** (0.741)	− 1.238*** (0.211)	− 1.424*** (0.323)	− 0.970 (0.585)	− 1.858*** (0.245)	− 1.882*** (0.358)	− 1.691* (0.662)	− 2.218*** (0.266)	− 2.507*** (0.389)	− 2.755*** (0.800)
**Model fit statistics**															
**Hosmer-Lemeshow goodness-of-fit test**															
Chi-squared statistic	4.87	1.87	4.93	11.38	12.89	15.46	0.67	8.25	9.14	0.78	5.49	3.52	4.70	7.41	3.88
df	3	8	8	3	8	8	3	8	8	3	8	8	3	8	8
*p*-value	0.1819	0.9848	0.7645	0.0098**	0.1157	0.0509	0.8796	0.4097	0.3305	0.8538	0.7039	0.8975	0.1950	0.4931	0.8680
**Fit measures to compare models**															
AIC	508.66	506.71	506.99	501.77	497.32	499.29	684.08	681.89	683.08	582.54	588.61	590.50	553.93	558.98	560.85
BIC	517.37	532.84	537.48	510.49	523.46	529.78	692.79	708.03	713.57	591.26	614.75	620.99	562.64	585.12	591.34
**Likelihood ratio test (Ref: full model)**															
Chi-squared statistic	9.95		1.72	12.45		0.04	10.19		0.81	1.93		0.11	2.95		0.13
df	4		1	4		1	4		1	4		1	4		1
*p*-value	0.0412*		0.1897	0.0143*		0.8466	0.0374*		0.3683	0.7485		0.7372	0.5657		0.7163

Coefficients are from single-level logistic regression models, *N* = 576 female students; robust standard errors are in parentheses

*** *p* < 0.001,

** *p* <0.01,

* *p* <0.05

**Table 3:** Results of logistic regression models predicting male students’ intentions to major in STEM fields

Variables	Biological sciences			Physical sciences			Math			Computer science			Engineering		
	Model 1	Model 2	Model 3	Model 4	Model 5	Model 6	Model 7	Model 8	Model 9	Model 10	Model 11	Model 12	Model 13	Model 14	Model 15
	Baseline	Full	Full + interaction	Baseline	Full	Full + interaction	Baseline	Full	Full + interaction	Baseline	Full	Full + interaction	Baseline	Full	Full + interaction
**Counter-stereotypical beliefs about scientists**	1.177** (0.409)	0.950* (0.431)	1.267 (1.117)	0.782* (0.382)	0.332 (0.409)	0.793 (1.118)	0.488 (0.301)	0.541 (0.317)	0.574 (0.866)	0.569 (0.295)	0.681* (0.312)	1.064 (0.888)	0.733** (0.282)	0.829** (0.300)	0.677 (0.781)
**Interaction**															
Beliefs × Latinx (ref = beliefs × Black)			− 0.369 (1.199)			− 0.530 (1.190)			− 0.037 (0.919)			− 0.432 (0.937)			0.176 (0.833)
**Controls**															
Latinx (ref = Black)		− 0.003 (0.323)	0.227 (0.823)		0.227 (0.321)	0.552 (0.807)		0.256 (0.261)	0.278 (0.595)		0.461 (0.261)	0.716 (0.617)		0.323 (0.240)	0.223 (0.529)
Mother’s highest level of education		0.340 (0.312)	0.341 (0.312)		0.364 (0.298)	0.366 (0.298)		0.125 (0.254)	0.125 (0.254)		0.247 (0.249)	0.248 (0.249)		− 0.227 (0.240)	− 0.227 (0.240)
Standardized 8th grade math test score		− 0.145 (0.122)	− 0.145 (0.122)		− 0.027 (0.118)	− 0.027 (0.118)		0.066 (0.095)	0.066 (0.095)		0.056 (0.093)	0.056 (0.093)		0.025 (0.089)	0.025 (0.089)
8th grade high self-perception of science performance		0.406 (0.269)	0.406 (0.269)		0.914*** (0.258)	0.914*** (0.258)		− 0.070 (0.210)	− 0.070 (0.210)		− 0.158 (0.207)	− 0.159 (0.207)		− 0.005 (0.197)	− 0.004 (0.197)
Constant	− 2.414*** (0.264)	− 2.533*** (0.406)	− 2.735*** (0.782)	− 2.048*** (0.238)	− 2.475*** (0.395)	− 2.765*** (0.776)	− 1.015*** (0.178)	− 1.254*** (0.307)	− 1.274* (0.569)	− 0.920*** (0.175)	− 1.349*** (0.306)	− 1.579** (0.593)	− 0.319* (0.163)	− 0.585* (0.281)	− 0.497 (0.504)
**Model fit statistics**															
**Hosmer-Lemeshow goodness-of-fit test**															
Chi-squared statistic	5.11	11.85	10.01	10.54	6.75	10.10	2.96	5.45	4.10	2.87	3.53	4.54	3.02	6.73	8.27
df	4	8	8	4	8	8	4	8	8	4	8	8	4	8	8
*p*-value	0.2760	0.1580	0.2644	0.0323*	0.5633	0.2583	0.5640	0.7082	0.8477	0.5802	0.8966	0.8054	0.5542	0.5660	0.4078
**Fit measures to compare models**															
AIC	431.83	434.02	435.93	470.48	462.38	464.18	664.93	671.23	673.23	686.36	689.79	691.58	734.49	738.53	740.48
BIC	440.39	459.68	465.86	479.03	488.04	494.12	673.48	696.89	703.16	694.92	715.45	721.51	743.05	764.19	770.42
**Likelihood ratio test (Ref: full model)**															
Chi-squared statistic	5.81		0.10	16.10		0.20	1.70		0.00	4.57		0.22	3.96		0.04
df	4		1	4		1	4		1	4		1	4		1
*p*-value	0.2139		0.7567	0.0029**		0.6533	0.7907		0.9674	0.3340		0.6424	0.4110		0.8334

Coefficients are from single-level logistic regression models, *N* = 532 male students; robust standard errors are in parentheses

*** *p* < 0.001,

** *p* < 0.01,

* *p* < 0.05

## Data Availability

Original data is not available for public access since the stipulation of publishing this data in institutional archives was not a part of the agreement with the school district where the data was collected. Please contact the corresponding author to obtain access to the data analysis documents for this article.

## Footnotes

1 We follow the common definition of STEM used in most national reports and research, which includes the fields of mathematics, natural sciences (e.g., biological sciences and physical sciences), engineering, and computer and information sciences (Chen, 2009). Additionally, while many studies on STEM inequality choose to focus exclusively on computer science and/or engineering fields given currently high job demands in these fields and their very low levels of female representation, we point out that students of color (including women of color) remain underrepresented in the biological sciences (NSB, NSF, 2019), and therefore, this field represents a continued area in need of more research and improvement regarding equity.

2 Interested readers might wonder how many students reported a value of 1 (or not at all likely) on the original scale. This ranged from 27 to 38%, depending on the field. Further, the percent of students who indicated a 1 on all five STEM fields was less than 10%.

3 To ensure that our results were robust to alternative specifications of the scale, we also created a scale that was the sum of the original survey items, transformed via Box-Cox to address skewness. This additive scale variable was very strongly correlated (*r* = 0.90) with the scale variable we use in our study, and results using this transformed scale variable in logistic regression models yielded comparable results as those presented here.

4 We initially performed multi-level models given the clustered nature of our data, but found that there was only significant variance at levels 2 and 3 for models predicting boys’ intentions to major in computer science. To be conservative, we first ran multi-level logistic regression models (with no level-2 or level-3 predictors) to account for the clustering at these levels for all outcomes (and both genders). Those results were comparable to results presented here. Additionally, we also examined models that included school-level control variables, such as school size and the proportion of students that are eligible for free or reduced lunch (as a proxy for school socioeconomic status). While one of these level-3 predictors was significant in the full model of some outcomes, we exclude level-3 control variables in our final models given that they did not significantly improve model fit, and our key results remained unchanged.

5 Additional analyses comparing counter-stereotypical views of scientists by race/ethnicity (pooled across gender) revealed that Black youth had a significantly higher mean on this scale than Latinx youth (*t* = 4.82, *p* < 0.001), and the difference was about two-fifths of a standard deviation.

## Appendix

**Table 4:** Correlations between independent variables

	1	2	3	4
1. Counter-stereotypical beliefs about scientists	1			
2. Latinx (ref = Black)	− 0.135***	1		
3. Mother’s highest level of education	0.112***	− 0.274***	1	
4. Standardized 8th grade math test score	− 0.015	− 0.017	− 0.031	1
5. 8th grade high self-perception of science performance	0.245***	− 0.144***	0.185***	−0.017

*** *p* < 0.001,

** *p* < 0.01,

* *p* < 0.05

## Abbreviations

AIC: Akaike information criterion
BIC: Bayesian information criterion
BSSS: Broadening science in school study
CFA: Confirmatory factor analysis
CFI: Comparative fit index
EFA: Exploratory factor analysis
RMSEA: Root mean square error of approximation
SRMR: Standardized root mean squared residual
STEM: Science, Technology, Engineering, and Mathematics

## References

[R1] Archer KJ, & Lemeshow S (2006). Goodness-of-fit test for a logistic regression model fitted using survey sample data. The Stata Journal, 6(1), 97–105. 10.1177/1536867X0600600106.

[R2] Bagozzi RP, & Yi Y (1988). On the evaluation of structural equation models. Journal of the academy of marketing science, 16(1), 74–94. 10.1007/BF02723327.

[R3] Bandura A, Barbaranelli C, Caprara GV, & Pastorelli C (2001). Self-efficacy beliefs as shapers of children’s aspirations and career trajectories. Child development, 72(1), 187–206. 10.1111/1467-8624.00273. 11280478

[R4] Beardslee DC, & O’Dowd DD (1961). The college-student image of the scientist. Science, 133(3457), 997–1001. 10.1126/science.133.3457.997. 17743790

[R5] Beasley MA, & Fischer MJ (2012). Why they leave: The impact of stereotype threat on the attrition of women and minorities from science, math, and engineering majors. Social Psychology of Education, 15(4), 427–448. 10.1007/s11218-012-9185-3.

[R6] Blanchard Kyte S, & Riegle-Crumb C (2017). Perceptions of the social relevance of science: exploring the implications for gendered patterns in expectations of majoring in STEM fields. Social Sciences, 6(1), 19. 10.3390/socsci6010019. PMC11177846 38883188

[R7] Bryk AS, & Raudenbush SW (1992). Hierarchical linear models: Applications and data analysis methods. Newbury Park, CA: Sage Publications.

[R8] Carli LL, Alawa L, Lee Y, Zhao B, & Kim E (2016). Stereotypes about gender and science: Women≠ scientists. Psychology of Women Quarterly, 40(2), 244–260. 10.1177/0361684315622645.

[R9] Chambers DW (1983). Stereotypic images of the scientist: The Draw-a-Scientist Test. Science education, 67(2), 255–265. 10.1002/sce.3730670213.

[R10] Chen X (2009). Students who study science, technology, engineering, and mathematics (STEM) in postsecondary education (NCES 2009–161). Washington, DC: National Center for Educational Statistics.

[R11] Cheryan S, Master A, & Meltzoff A (2015). Cultural stereotypes as gatekeepers: Increasing girls’ interest in computer science and engineering by diversifying stereotypes. Frontiers in Psychology, 6(49), 1–8. 25717308 10.3389/fpsyg.2015.00049 PMC4323745

[R12] Cheryan S, Plaut VC, Handron C, & Hudson L (2013). The stereotypical computer scientist: Gendered media representations as a barrier to inclusion for women. Sex roles, 69(1–2), 58–71. 10.1007/s11199-013-0296-x.

[R13] Cheryan S, Ziegler SA, Montoya AK, & Jiang L (2017). Why are some STEM fields more gender balanced than others? Psychological Bulletin, 143(1), 1–35. 10.1037/bul0000052. 27732018

[R14] Christidou V, Bonoti F, & Kontopoulou A (2016). American and Greek children’s visual images of scientists. Science & Education, 25(5–6), 497–522. 10.1007/s11191-016-9832-8.

[R15] Collins PH (1998). It’s all in the family: Intersections of gender, race, and nation. Hypatia, 13(3), 62–82. 10.1111/j.1527-2001.1998.tb01370.x.

[R16] Costello AB, & Osborne J (2005). Best practices in exploratory factor analysis: Four recommendations for getting the most from your analysis. Practical assessment, research, and evaluation, 10(7), 1–9.

[R17] de Brey C, Musu L, McFarland J, Wilkinson-Flicker S, Diliberti M, Zhang A, … Wang X (2019). Status and Trends in the Education of Racial and Ethnic Groups 2018 (NCES 2019–038). U.S. Department of Education. Washington, DC: National Center for Education Statistics Retrieved 5 January 2020 from https://nces.ed.gov/pubsearch/.

[R18] DeCoster J (1998). Overview of Factor Analysis. Retrieved August 13, 2020 from http://www.stat-help.com/notes.html

[R19] DeWitt J, Archer L, Osborne J, Dillon J, Willis B, & Wong B (2011). High aspirations but low progression: The science aspirations–careers paradox amongst minority ethnic students. International Journal of Science and Mathematics Education, 9(2), 243–271. 10.1007/s10763-010-9245-0.

[R20] DeWitt J, Osborne J, Archer L, Dillon J, Willis B, & Wong B (2013). Young children’s aspirations in science: The unequivocal, the uncertain and the unthinkable. International Journal of Science Education, 35(6), 1037–1063. 10.1080/09500693.2011.608197.

[R21] Diekman A, Steinberg M, Brown E, Belanger A, & Clark E (2017). A Goal Congruity Model of Role Entry, Engagement, and Exit: Understanding Communal Goal Processes in STEM Gender Gaps. Personality and Social Psychology Review, 21(2), 142–175. 10.1177/1088868316642141. 27052431

[R22] Diekman AB, Brown ER, Johnston AM, & Clark EK (2010). Seeking congruity between goals and roles: A new look at why women opt out of science, technology, engineering, and mathematics careers. Psychological science, 21(8), 1051–1057. 10.1177/0956797610377342. 20631322

[R23] Eccles JS, & Roeser RW (2011). Schools as developmental contexts during adolescence. Journal of research on adolescence, 21(1), 225–241. 10.1111/j.1532-7795.2010.00725.x.

[R24] Ehrlinger J, Plant EA, Hartwig MK, Vossen JJ, Columb CJ, & Brewer LE (2018). Do gender differences in perceived prototypical computer scientists and engineers contribute to gender gaps in computer science and engineering? Sex roles, 78(1–2), 40–51. 10.1007/s11199-017-0763-x. 29367799 PMC5756563

[R25] Else-Quest NM, Mineo CC, & Higgins A (2013). Math and science attitudes and achievement at the intersection of gender and ethnicity. Psychology of Women Quarterly, 37(3), 293–309. 10.1177/0361684313480694.

[R26] Engberg ME, & Wolniak GC (2013). College student pathways to the STEM disciplines. Teachers College Record, 115(1), 1–27.

[R27] Erb TO, & Smith WS (1984). Validation of the attitude toward women in science scale for early adolescents. Journal of Research in Science Teaching, 21(4), 391–397. 10.1002/tea.3660210407.

[R28] Fabrigar LR, Wegener DT, MacCallum RC, & Strahan EJ (1999). Evaluating the use of exploratory factor analysis in psychological research. Psychological methods, 4(3), 272–299. 10.1037/1082-989X.4.3.272.

[R29] Farland-Smith D (2009a). Exploring middle school girls’ science identities: Examining attitudes and perceptions of scientists when working “side-by-side” with scientists. School Science and Mathematics, 109(7), 415–427. 10.1111/j.1949-8594.2009.tb17872.x.

[R30] Farland-Smith D (2009b). How does culture shape students’ perceptions of scientists? Cross-national comparative study of American and Chinese elementary students. Journal of Elementary Science Education, 21(4), 23–42. 10.1007/BF03182355.

[R31] Finson KD (2003). Applicability of the DAST-C to the images of scientists drawn by students of different racial groups. Journal of Elementary Science Education, 15(1), 15–26. 10.1007/BF03174741.

[R32] Finson KD, Beaver JB, & Cramond BL (1995). Development and field test of a checklist for the Draw-A-Scientist Test. School Science and Mathematics, 95(4), 195–205. 10.1111/j.1949-8594.1995.tb15762.x.

[R33] Fraser BJ (1978). Development of a test of science-related attitudes. Science Education, 62(4), 509–515. 10.1002/sce.3730620411.

[R34] Fung YY (2002). A comparative study of primary and secondary school students’ images of scientists. Research in Science & Technological Education, 20(2), 199–213. 10.1080/0263514022000030453.

[R35] Garriott PO, Hultgren KM, & Frazier J (2017). STEM stereotypes and high school students’ math/science career goals. Journal of Career Assessment, 25(4), 585–600. 10.1177/1069072716665825.

[R36] Garrison H (2013). Underrepresentation by race–ethnicity across stages of US science and engineering education. CBE—Life Sciences Education, 12(3), 357–363. 10.1187/cbe.12-12-0207. 24006384 PMC3763003

[R37] Hair JF, Black WC, Babin BJ, & Anderson RE (2010). Multivariate data analysis, (7th ed., ). Upper Saddle River, NJ: Prentice-Hall, Inc.

[R38] Hanson SL (2004). African-American women in science: Experiences from high school through the post-secondary years and beyond. NWSA Journal, 16(1), 96–115. 10.2979/NWS.2004.16.1.96.

[R39] Hillman SJ, Bloodsworth KH, Tilburg CE, Zeeman SI, & List HE (2014). K-12 Students’ Perceptions of Scientists: Finding a valid measurement and exploring whether exposure to scientists makes an impact. International Journal of Science Education, 36(15), 2580–2595. 10.1080/09500693.2014.908264.

[R40] Hu LT, & Bentler PM (1999). Cutoff criteria for fit indexes in covariance structure analysis: Conventional criteria versus new alternatives. Structural equation modeling: a multidisciplinary journal, 6(1), 1–55. 10.1080/10705519909540118.

[R41] Ireland DT, Freeman KE, Winston-Proctor CE, DeLaine KD, McDonald Lowe S, & Woodson KM (2018). (un) hidden figures: A synthesis of research examining the intersectional experiences of black women and girls in STEM education. Review of Research in Education, 42(1), 226–254. 10.3102/0091732X18759072.

[R42] Kaiser HF (1974). An index of factorial simplicity. Psychometrika, 39(1), 31–36. 10.1007/BF02291575.

[R43] Kline RB (2015). Principles and practice of structural equation modeling. New York, NY: Guilford publications.

[R44] Laubach TA, Crofford GD, & Marek EA (2012). Exploring Native American students’ perceptions of scientists. International Journal of Science Education, 34(11), 1769–1794. 10.1080/09500693.2012.689434.

[R45] Lichtenberger E, & George-Jackson C (2013). Predicting high school students’ interest in majoring in a STEM field: Insight into high school students’ postsecondary plans. Journal of Career and Technical Education, 28(1), 19–38.

[R46] Long JS, & Freese J (2006). Regression models for categorical dependent variables using Stata. College Station, TX: Stata press.

[R47] Maerten-Rivera J, Myers N, Lee O, & Penfield R (2010). Student and school predictors of high-stakes assessment in science. Science Education, 94(6), 937–962. 10.1002/sce.20408.

[R48] Maple SA, & Stage FK (1991). Influences on the choice of math/science major by gender and ethnicity. American Educational Research Journal, 28(1), 37–60. 10.3102/00028312028001037.

[R49] Miller DI, Nolla KM, Eagly AH, & Uttal DH (2018). The development of children’s gender-science stereotypes: a meta-analysis of 5 decades of US draw-a-scientist studies. Child development, 89(6), 1943–1955. 10.1111/cdev.13039. 29557555

[R50] Monhardt RM (2003). The image of the scientist through the eyes of Navajo children. Journal of American Indian Education, 42(3), 25–39.

[R51] Morgan SL, Gelbgiser D, & Weeden KA (2013). Feeding the pipeline: Gender, occupational plans, and college major selection. Social Science Research, 42(4), 989–1005. 10.1016/j.ssresearch.2013.03.008. 23721669

[R52] Morris LK, & Daniel LG (2008). Perceptions of a chilly climate: Differences in traditional and non-traditional majors for women. Research in Higher Education, 49(3), 256–273. 10.1007/s11162-007-9078-z.

[R53] Nassar-McMillan SC, Wyer M, Oliver-Hoyo M, & Schneider J (2011). New tools for examining undergraduate students’ STEM stereotypes: Implications for women and other underrepresented groups. New Directions for Institutional Research, 2011(152), 87–98. 10.1002/ir.411.

[R54] National Science Board, National Science Foundation (2019). Higher Education in Science and Engineering. Science and Engineering Indicators 2020. NSB-2019–7. Alexandria, VA: Available at https://ncses.nsf.gov/pubs/nsb20197/.

[R55] Newton LD, & Newton DP (1998). Primary children’s conceptions of science and the scientist: is the impact of a National Curriculum breaking down the stereotype? International Journal of Science Education, 20(9), 1137–1149. 10.1080/0950069980200909.

[R56] O’Brien LT, Blodorn A, Adams G, Garcia DM, & Hammer E (2015). Ethnic Variation in Gender-STEM Stereotypes and STEM Participation: An Intersectional Approach. Cultural Diversity and Ethnic Minority Psychology, 21(2), 169–180. 10.1037/a0037944. 25244590

[R57] Ong M, Smith JM, & Ko LT (2018). Counterspaces for women of color in STEM higher education: Marginal and central spaces for persistence and success. Journal of Research in Science Teaching, 55(2), 206–245. 10.1002/tea.21417.

[R58] Ong M, Wright C, Espinosa L, & Orfield G (2011). Inside the Double Bind: A Synthesis of Empirical Research on Undergraduate and Graduate Women of Color in Science, Technology, Engineering, and Mathematics. Harvard Educational Review, 81(2), 172–209. 10.17763/haer.81.2.t022245n7×4752v2.

[R59] Rainey K, Dancy M, Mickelson R, Stearns E, & Moller S (2018). Race and gender differences in how sense of belonging influences decisions to major in STEM. International Journal of STEM Education, 5(1), 10. 10.1186/s40594-018-0115-6. 30631700 PMC6310405

[R60] Ridolfo H, & Maitland A (2011). Factors that influence the accuracy of adolescent proxy reporting of parental characteristics: A research note. Journal of adolescence, 34(1), 95–103. 10.1016/j.adolescence.2010.01.008. 20153517

[R61] Riegle-Crumb C, & King B (2010). Questioning a white male advantage in STEM: Examining disparities in college major by gender and race/ethnicity. Educational Researcher, 39(9), 656–664. 10.3102/0013189X10391657.

[R62] Riegle-Crumb C, & Morton K (2017). Gendered expectations: Examining how peers shape female students’ intent to pursue STEM fields. Frontiers in psychology, 8, 329. 28360868 10.3389/fpsyg.2017.00329 PMC5350122

[R63] Schibeci RA (1986). Images of science and scientists and science education. Science Education, 70(2), 139–149. 10.1002/sce.3730700208.

[R64] Schinske J, Cardenas M, & Kaliangara J (2015). Uncovering scientist stereotypes and their relationships with student race and student success in a diverse, community college setting. CBE—Life Sciences Education, 14(3), ar35. 26338318 10.1187/cbe.14-12-0231 PMC4710393

[R65] Shapiro JR, & Williams AM (2012). The role of stereotype threats in undermining girls’ and women’s performance and interest in STEM fields. Sex Roles, 66(3–4), 175–183. 10.1007/s11199-011-0051-0.

[R66] Shin SY, Parker LC, Adedokun O, Mennonno A, Wackerly A, & San Miguel S (2015). Changes in elementary student perceptions of science, scientists, and science careers after participating in a curricular module on health and veterinary science. School science and mathematics, 115(6), 271–280. 10.1111/ssm.12129. 26726271 PMC4696504

[R67] Smith WS, & Erb TO (1986). Effect of women science career role models on early adolescents’ attitudes toward scientists and women in science. Journal of Research in Science Teaching, 23(8), 667–676. 10.1002/tea.3660230802.

[R68] Song J, & Kim KS (1999). How Korean students see scientists: the images of the scientist. International Journal of Science Education, 21(9), 957–977. 10.1080/095006999290255.

[R69] Starr CR (2018). “I’m Not a Science Nerd!” STEM Stereotypes, Identity, and Motivation Among Undergraduate Women. Psychology of Women Quarterly, 42(4), 489–503. 10.1177/0361684318793848.

[R70] Steinke J, Lapinski MK, Crocker N, Zietsman-Thomas A, Williams Y, Evergreen SH, & Kuchibhotla S (2007). Assessing media influences on middle school–aged children’s perceptions of women in science using the Draw-A-Scientist Test (DAST). Science Communication, 29(1), 35–64. 10.1177/1075547007306508.

[R71] Supeli A, & Creed P (2014). The Incremental Validity of Perceived Goal Congruence: The Assessment of Person-Organizational Fit. Journal of Career Assessment, 22(1), 28–42. 10.1177/1069072713487849.

[R72] Thomas MD, Henley TB, & Snell CM (2006). The draw a scientist test: A different population and a somewhat different story. College Student Journal, 40(1), 140–149.

[R73] Wang J (2005). Relationship Between Mathematics and Science Achievement at the 8th Grade. Online Submission, 5, 1–17.

[R74] Wang X (2013). Why students choose STEM majors: Motivation, high school learning, and postsecondary context of support. American Educational Research Journal, 50(5), 1081–1121. 10.3102/0002831213488622.

[R75] Wyer M (2003). Intending to stay: Images of scientists, attitudes toward women, and gender as influences on persistence among science and engineering majors. Journal of Women and Minorities in Science and Engineering, 9(1), 1–16. 10.1615/JWomenMinorScienEng.v9.i1.10.

[R76] Wyer M, Schneider J, Nassar-McMillan S, & Oliver-Hoyo M (2010). Capturing stereotypes: Developing a scale to explore US college students’ images of science and scientists. International Journal of Gender, Science and Technology, 2(3), 381–415.

[R77] Xie Y, Fang M, & Shauman K (2015). STEM education. Annual review of sociology, 41(1), 331–357. 10.1146/annurev-soc-071312-145659. PMC4712712 26778893

